# New Forms of Electrospun Nanofibers Applied in Cardiovascular Field

**DOI:** 10.3389/fcvm.2021.801077

**Published:** 2022-01-21

**Authors:** Weimin Huang, Mengen Huo, Nan Cheng, Rong Wang

**Affiliations:** ^1^Baotou Clinical Medical College, Inner Mongolia Medical University, Hohhot, China; ^2^Department of Cardiac Surgery, Chinese PLA General Hospital, Beijing, China; ^3^Institute of Poisons and Drugs, Beijing Academy of Military Medical Sciences, Beijing, China

**Keywords:** electrospinning (ES), nanofibers (NFs), cardiac patch, vascular transplantation, tissue engineering

## Abstract

Cardiovascular disease (CVD) is one of the leading causes of death worldwide. In recent years, regenerative medicine, tissue engineering and the development of new materials have become the focus of attention this field, and electrospinning technology to prepare nanofibrous materials for the treatment of cardiovascular diseases has attracted people's attention. Unlike previous reviews, this research enumerates the experimental methods and applications of electrospinning technology combined with nanofibrous materials in the directions of myocardial infarction repair, artificial heart valves, artificial blood vessels and cardiovascular patches from the perspective of cardiovascular surgery. In the end, this review also summarizes the limitations, unresolved technical challenges, and possible future directions of this technology for cardiovascular disease applications.

## Introduction

In 1985, Weinstein and Stason (1) have reported that coronary heart disease (CHD) is the leading cause of death and disability in the United States. One in five Americans develops CHD on their sixtieth birthday. Of these, 11% did die suddenly and another 44% suffered a non-fatal myocardial infarction, with an economic burden of well-over $100 billion.

In today's society, cardiovascular disease is still the leading cause of death throughout the world, causing more than 17.9 million deaths each year (2). According to the American Heart Association (AHA) estimates, by 2035, 46.1% of the US population will have some form of cardiovascular disease, when the total cost associated with cardiovascular disease will be 1.1 trillion dollars (3), which is enough to see the great harm and burden of cardiovascular disease to humans.

Myocardial infarction is usually due to the lack of oxygen and nutrients in the myocardium, resulting in the death of cardiomyocytes (4). The myocardial tissue after infarction lacks the ability of regeneration. Implanting tissue-engineered myocardium into diseased heart seems to be the simplest way to repair infarcted myocardium. At present, the limitations of myocardial tissue regeneration include the inability to fully summarize the structure and mechanical environment of natural heart tissue (5). Scaffolds made of electrospinning nanofibers have been actively explored for myocardial tissue regeneration.

**Figure 1:**
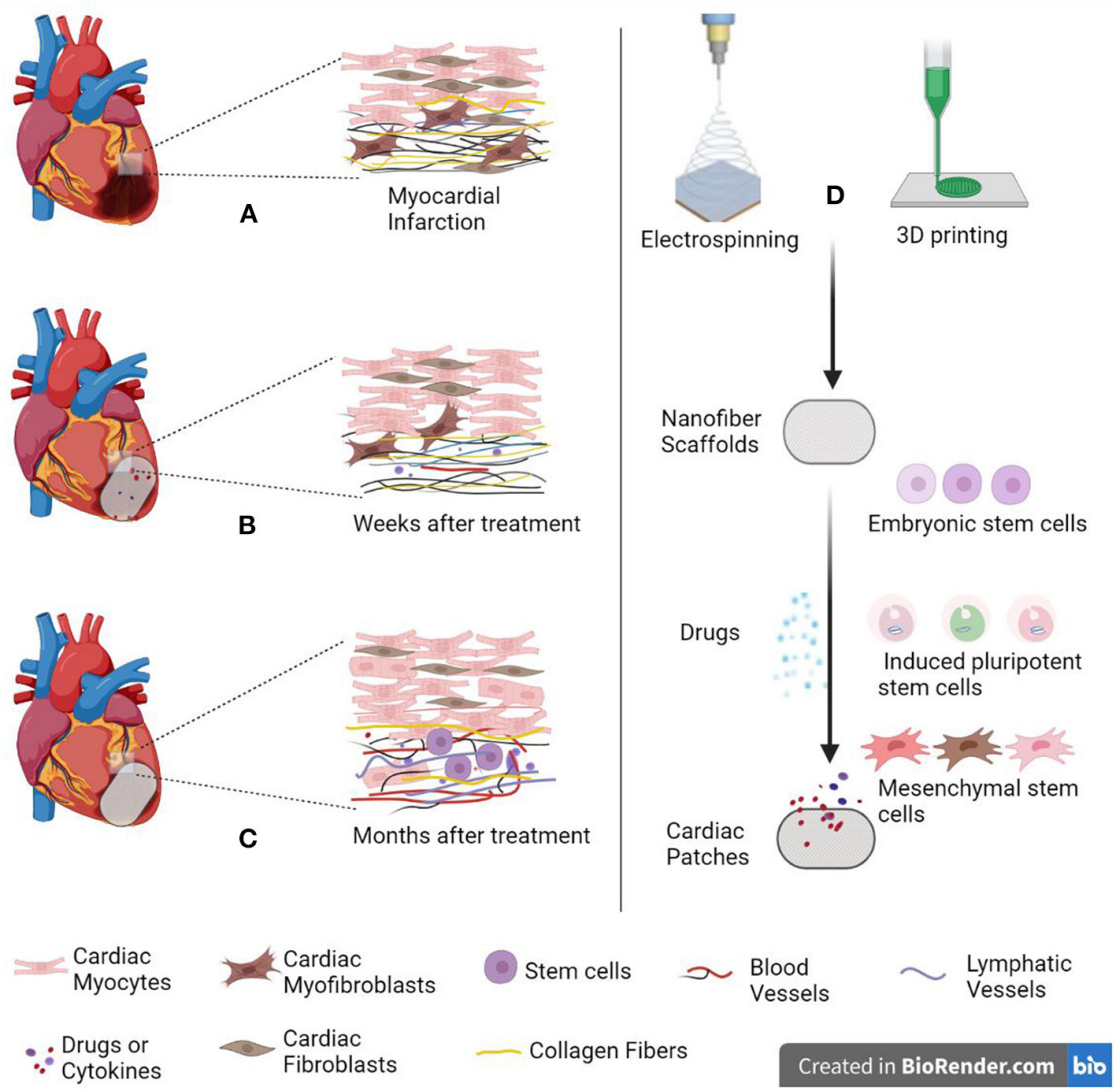
Schematic diagram of cardiac patch repairing myocardial infarction. **(A)** When myocardial infarction occurs, collagen fibers proliferate and form scar tissue. **(B)** Several weeks after treatment. **(C)** Several months after treatment. **(D)** Cardiac patch is made by electrospinning combined with 3D printing technology. The patch simulates extracellular matrix (ECM) to provide mechanical support for cells. Created with BioRender.com.

As shown in the Figure 1, in order to improve the survival rate of stem cells in repairing myocardial injury, electrospinning scaffolds are used to mechanically support and mimic extracellular matrix structure to improve cell adhesion, viability, and regeneration (6).

More than 50% of all deaths caused by CVD are attributed to vascular injury induced by vascular plaque aggregation, which leads to vascular obstruction and sclerosis (7). People have been trying to develop appropriate alternative vessels from autologous vascular transplantation, allografts and synthetic grafts. However, these sources could not meet the needs of alternative vessels, especially the long-term patency rate of small-diameter vascular transplantation has always been a difficult problem in the world (8).

For more than 50 years, conventional coronary artery bypass grafting (CABG) has been the gold standard for the treatment of coronary heart disease (9). There are hundreds of thousands of coronary artery bypass procedures in the world each year, for small diameter grafts (<6 mm), these synthetic grafts often failed due to rapid occlusion and thrombosis, so there is still no effective alternative to autologous vascular grafting (10, 11).

The patency and biocompatibility of small-diameter vascular grafts synthesized by traditional tissue engineering strategies are poor (32), and their clinical transformation is seriously limited by high cost and long production time (20). In recent years, tissue engineering using rapidly degradable materials can induce the regeneration potential of the host, and summarize natural tissue regeneration through reasonable graft design, including structural optimization (30, 33) and functionalization (5, 34).

It is not only required that the small-diameter vascular stent should have the characteristics of maintaining the blood flow in the lumen without leakage, but also that it should have anticoagulant and antithrombotic ability in function to prevent stenosis and occlusion (35). Therefore, many researchers are engaged in electrospinning technology to prepare grafts with different materials, and study the methods to promote the proliferation of vascular intimal endothelial cells and control the proliferation of smooth muscle cells (SMCs). The general method is to fold the electrospinning membrane into a tubular structure through a rolling rod collector, and load heparin (22), growth factors (36) and other active substances (37) on its surface for the study of vascular tissue regeneration. As shown in the Table 1, Animal models, included rat abdominal aorta (19, 20, 29), rabbit carotid artery (21–23, 29), sheep carotid artery (31) and canine femoral artery (38), are often used to test the performance of vascular stents, ranging from several weeks to months.

**Table 1:** Construction strategies and *vivo/vitro* experiments of electrospun fiber membrane in the reviews.

**Nanofiber materials**	**Diameter/Aperture**	**Thickness (μm)**	**Drugs or cells**	**Animals**	**Experiments**	**Time (weeks)**	**References**
PCL	Inner: 300 ± 100 nm Out: 2.8 ± 0.13 μm	100 μm	Cardiomyocytes /fibroblasts	Mice	*In vitro*	1	(12)
PLGA	N/A	110 ± 10μm	Endothelial cells, VEGF granules, dexamethasone	Mice	Cardiac patch	2	(13)
PCL-Gelatin	578 ± 184 nm	115 ± 11 μm	hiPSC-CMs	N/A	*In vitro*	2	(6)
PCL	200–5,500 nm	50 μm	Bone marrow and heart stem cells	Mice	LAD in rats (suture)	3	(14)
β-PVDF	N/A	N/A	TiO2	N/A	*In vitro*	6^*^	(15)
AuNRs	500 nm/20–60 nm	60–80/100–120 μm	Left ventricular cardiomyocytes	Mice	Cardiac patch (Near IR)	1	(16)
PCL/GelMA-Ppy nanoparticles	948 ± 153 nm	N/A	Cardiomyocytes/fibroblasts	Mice	Cardiac patch 1.5 ×1.5cm	4	(17)
PCL/NO	690 nm/3.4 μm	600 μm	NO_2_	Mice	Cardiac patch 0.4 ×0.6cm	4	(18)
PCL/Heparin coating	-/21.2 ± 0.79 μm	295 ± 5.52 μm	Heparin	Mice	Aortic replacement	3	(19)
PLCL	6 μm/300 nm	500 μm	Hyaluronan	Mice	Aortic replacement	24	(20)
PELCL/chitosan-hydrogel	Inner: 754 ± 385 nm Out: 1,087 ± 526 nm	N/A	Inner: VEGF Out: PDGF	Rabbits	Carotid artery transplantation	4	(21)
PLCL	821 ± 102.87 nm	300 ± 17 μm	Heparin / Silk Fibroin	Rabbits	Carotid artery transplantation	32	(22)
PCL	263.1 ± 90.2 nm	N/A	KSNO	Rabbits	Carotid artery transplantation	4	(23)
PLA-PCL	N/A	40 ± 7/175 ± 4 μm	Human fibroblast	N/A	*In vitro*	8	(24)
ADF4(C16)	1.6 ± 0.2 μm	N/A	N/A	Mice	Arteriovenous loop model	4	(25)
ESM/TPU	435.86 ± 173.27 nm	50–70 μm	HUVEC	N/A	*In vitro*	1	(26)
CS-PVA-CNT	255 ± 3.5 nm	N/A	MSCs	N/A	*In vitro*	3	(11)
CS/PLCL	110.09 ± 16.33nm	50 μm	Dextran Sulfate	Mice	*In vivo*	4	(27)
SF/TPU	N/A	100–500 μm	SF (Silk Fibroin)	Dogs	Aortic wall repair 2 ×1 cm	12	(28)
SF/PU	1.32 ± 0.78 μm	100 μm	SF (Silk Fibroin)	Mice	Aortic wall repair, 0.3 ×0.6 cm	24	(29)
PLA/PCL PU/PCL	N/A	15.47 ± 1.31 μm 127.87 ± 2.38 μm 19.96 ± 1.18 μm	HUVCEs/VSMCs	N/A	*In vitro* evaluation, 0.6 cm	1	(30)
PCL/collagen scaffolds	4.45 ± 0.81 μm	400 μm	ECs/SMCs	Sheep	Carotid artery, 4.75 mm ×5 cm	24	(31)
PELCL/chitosan hydrogel	754 ± 385 nm 1,124 ± 529 nm	N/A	VEGF/PDGF	Rabbits	Carotid artery, 2.2mm	4	(21)

*PCL, polycaprolactone; PLCL, poly(L-Lactide-co-caprolactone); PLGA, poly-lactic-co-glycolic acid; β-PVDF, β-polyvinylidene fluoride; AuNRs, albumin electrospinning fibers and gold nanorods; GelMA-Ppy, methacrylic anhydride-gelatin-polypyrrole; PELCL, poly-(ethylene glycol)-b-poly(L-lactide-co-caprolactone); PLA-PCL, poly-L-lactide-co-poly-ε-caprolactone; KSNO, NO donor of S-nitrosated keratin; ADF4(C16), one of the spider silk protein; ESM/TPU, double-layered eggshell membrane/thermoplastic polyurethane; CS-PVA-CNT, chitosan, polyvinyl alcohol, carbon nanotube; HUVEC, human umbilical vein endothelial cell; MSCs, mesenchymal stem cells; VEGF, vascular endothelial growth factor; PDGF, platelet-derived growth factor; ECs, endothelial cells; SMCs, smooth cells*.

“*” *The symbol represents the unit of data mentioned in the literature as hours, not weeks. The “N/A” symbol is not available which means that the data cannot be extracted or not mentioned in this article*.

With the development of tissue engineering technologies in recent years, a series of nanofibrous materials based on electrospinning technology have attracted much attention of researchers (39).

In the field of cardiovascular tissue regeneration, compared with traditional regeneration technology, electrospinning technology has the following advantages:

1. The cardiac patches loaded with stem cells can repair the infarcted myocardial region without limiting the later cardiac systolic activity.
2. Simulating the unique physical structure of natural cardiac leaflets, the leaflet structure with the same anisotropy and mechanical strength is provided.
3. The vascular scaffold can be used to match the proliferation of vascular endothelial cells with the degradation of the material, so as to ensure a higher patency rate.
4. It can provide intelligent health monitoring function, which can early evaluate and prevent acute and chronic cardiovascular diseases.

In this study, we would focus on nanofibers, introduce the progress of different electrospinning technologies in the field of heart and vessel-related tissue engineering, compare the effect of some nanofiber materials, and forecast the development of electrospinning technology in the field of cardiovascular medicine and the problems to be broken through.

### Myocardial Infarction Repair

From the microstructure,Sharon Fleischer (12) divided the myocardium into three fiber groups with specific effect and different sizes: nanoscale endomysial fibers, perimysial fibers with a diameter of 1 μm and epimysium fibers with a diameter of several microns. He also reported that the electrospinning fiber stent with spring-like coiled fiber structure was conducive to cardiac tissue engineering. Later, the team (13) designed a cage structure with microgrooves and sidewall microchannel albumin stent to arrange the cardiac tissue and accommodate the growth of endothelial cells. This cage structure can also accommodate the particle system, control the release of VEGF, promote vascularization, and even load dexamethasone drugs to achieve the effect. In addition, Zhu et al. (18) mixed the polymer caprolactone with caprolactone NO_2_ to prepare nitric acid-functionalized cardiac patches to implant the site of myocardial infarction using the electrospinning method. The results showed that NO was gradually released from the patch under ischemic microenvironment, and the effect of NO patch group was significant. What attracts our attention is an aligned polycaprolactone (PCL) -Gelatin coaxial nanofiber patch was fabricated by Kuma (6) using electrospinning. The results show that cells on cardiac patches exhibit synchronous contraction and exhibit a rapid response to cardiac drugs. The patches could be scaled to serve as an *in vitro* drug screening platform for cardiotoxicity studies.

Despite some success with cell-loading techniques, many scaffolds have limited cell infiltration and low cell survival (40). In order to overcome the limited cell infiltration of cardiac patches and also consider the supporting mechanical properties of these patches, Chen and Kan (14) designed the thickness of the patches to about 50 μm. Both bone marrow and human cardiac stem cells cultured on these patches had good survival and infiltration (~30 μm). In addition, the tensile strength of the patch could withstand the severe pumping effect of myocardium, confirming that the material prepared by this method has the potential to be used as a scaffold for cardiomyocyte repair and application. Unlike the above ideas, the glycosaminoglycan (GAG) mimetic peptide nanofiber gel synthesized by Rufaihah et al. (41) was injected into the infarct site, emphasizing its approach of repairing the myocardium and inducing neovascularization without adding any biologically derived factors or stem cells. This predicts the possibility of electrospinning technology combined with nanofibrous hydrogels in tissue engineering.

In order to avoid the problem of additional damage caused by suturing the patch to the heart using surgical means in the past, Malki et al. (16) developed a nanocomposite scaffold composed of albumin electrospinning fibers and gold nanorods (AuNRs), which was positioned and irradiated with a near-infrared laser (808 nm), and AuNRs were able to absorb light and convert it into thermal energy, locally change the molecular structure of the fibrous scaffold, eventually attach it strongly but safely to the heart wall. Subsequently, He et al. (17) were inspired by mussels to design conductive nanofibrous membranes to repair myocardial infarction by enhancing cardiac function and revascularization. The result showed that 4 weeks after patch transplantation on the infarcted heart, the infarct size was reduced by about 50%, the percentage of left ventricular fraction was increased by about 20%, and the density of neovascularization in the infarcted area was significantly increased by about 9-fold compared with the control group.

In order for the patches to exhibit mechanical and conductive properties similar to those of autologous myocardium, Walker et al. (42) developed cardiac gaskets, first of which was gelatin methacryloyl (GelMA), followed by highly adhesive fibrous scaffolds modified with conjugated choline-based biological ionic liquids (bio-IL), based on the formation of ionic bonds between Bio-IL and native tissue, and the engineered patches adhered strongly to rat myocardium without suturing.

In order to establish a model for evaluating the performance of engineered heart *in vitro*, Polylactic acid (PLA) and poly-ε-caprolactone (PCL) were used to fabricate the porous scaffold via 3D printing and electrospinning, and then cardiomyocytes from neonatal Sprague Dawley (SD) rats were cultured on the PLA/PCL scaffold to construct the engineered cardiac tissue (ECT). The strength and biocompatibility of the scaffold were verified via evaluating the cell viability and mechanical beating status (43). This work provides a new approach for evaluating ECT, which is expected to be applied to pharmaceutical studies.

### Tissue Engineering of Heart Valves

At present, biological valves also have some limitations, especially in young patients, so it is critical to continue to develop new materials. But heart valve tissue engineering still faces challenges, for example, the valve component has three layers, which are circumferential, random and radial, respectively, which increases the difficulty of material preparation. Wu et al. (44) began to believe that the hydrogel system of methacrylic hyaluronic acid (Me-HA) and methacrylate gelatin (Me-Gel) mixed with each other can simulate the unique 3D physiological microenvironment of the ECM of native aortic valve leaflets, but it was later found that these hydrogel materials lacked macroscopic anisotropic structure and had weak extension ability, so it was thought to compensate for the disadvantages of hydrogels by adding fibrous components.

In order to better simulate the direction, Jana and Lerman (45) from Mayo Clinic firstly designed three new collectors to fabricate three nanofiber layers with these directions from polymeric biomaterials in electrospinning systems.

### Cardiac Surgeries

In order to develop a new generation of biomaterials for closure of atrial septal defect, Kaiser firstly designed chamber-deficient patches using medical grade polyurethane loaded with bioactive agents chitosan nanoparticles and collagen, followed by coating with heparin (46). In the postoperative aspect of cardiac surgery, sternal and epicardial adhesions increase the risk and complexity of cardiac reoperation, which is a significant challenge for the later rehabilitation of all patients who underwent cardiac surgery again. Feng et al. (47) fabricated a bioabsorbable GT/PCL composite membrane to prevent adhesions in cardiac surgery in a rabbit model and proposed its use as a novel pericardial substitute for cardiac surgery.

### Small-Caliber Vascular Grafts

At present, there are practical non-tissue engineered grafts for large vessels in clinical practice, but doctors often face failure in front of small-caliber vascular grafts due to the easy thrombosis. To prevent thrombosis, Zhang et al. (21) developed two modified coaxial electrospinning techniques, which are to achieve vascular compliance. Interestingly, previous studies have been observed in vascular transplantation experiments for a maximum of 3 months (19), while Qin et al. (20) reported the time up to 6 months. They agreed that the vascular smooth muscle layer is essential for maintaining the mechanical strength and vasoactive reactivity of blood vessels, so hyaluronic acid was loaded on rapidly biodegradable vascular grafts in the study and concluded that it promotes vascular smooth muscle regeneration.

However, Kuang et al. (22) considered that smooth muscle cells (SMCs) are prone to excessive proliferation and cause restenosis at the late stage of implantation. In order to develop a safe and unobstructed artificial blood vessel, they prepared a small-caliber artificial blood vessel with composite nanofiber nucleocapsid structure by a combination of conjugated electrospinning and lyophilization technology. The inner layer provides mechanical support during vascular reconstruction. The shell, heparin/silk fibroin layer, enhances the biocompatibility of the graft, and the release of heparin at the early stage after transplantation can regulate the microenvironment, promote endothelial cell growth and inhibit smooth muscle proliferation. This animal experiment showed that the graft patency time remained more than 8 months, which far exceeded 3 months which Wu has reported (19).

In order to better promote the endo-thelialization of transplanted vessels and simulate the extracellular matrix, hyaluronic acid oligosaccharide-modified collagen was fabricated into nanofibers by electrospinning technique in Kang et al. (48) from Shandong University. The *in vitro* experimental results supported that it promoted endothelial cell proliferation and had antithrombotic properties. Lee et al. (39) of Seoul University encapsulated human ASC spheres in alginate-based scaffolding structures by a combined 3D printing/electrospinning system. In order to treat peripheral arterial occlusive disease, Dorati preliminarily explored the replacement of artificial blood vessels in damaged peripheral arteries, which showed that the electrospinning technique was suitable for obtaining grafts <6 mm in diameter and between 140 ± 7 and 175 ± 4 μm in thickness. Finally, vascular grafts with the best mechanical properties similar to natural bovine blood vessels were designed (24).

In order to reduce the toxicity of transplanted blood vessels, Li et al. (23) synthesized a low-toxic NO donor of S-nitrosated keratin (KSNO) and then co-electrospinning with poly-ε-caprolactone to obtain NO-releasing small-diameter vascular grafts. In order to improve the biocompatibility of transplantation, Yan et al. (26) reported a wavy structure of small-diameter, double-layered eggshell membrane/thermoplastic polyurethane (ESM/TPU) vascular graft.

It is not easy to maintain good supporting performance of transplanted blood vessels and make their cell adhesion strong. To obtain vascular graft with stronger mechanical properties and cell-guided growth ability, Liu has developed a biomimetic three-layer vascular graft with strong mechanical properties and cell-guided growth ability (30).

Of course, the strategy of vascular tissue engineering is eventually to combine autologous vascular cells with tubular biodegradable scaffolds, and Ju et al. did manufacture vascular substitutes (31). This study demonstrates that electrospinning double-layer vascular scaffolds combined with autologous vascular cells may be a clinically applicable alternative to conventional graft substitutes.

### Vascular Patches

Vascular patches currently used in cardiovascular surgery have several disadvantages, including material degeneration, calcification, and pseudointimal hyperplasia leading to hemodynamic disturbances (28). Initially Chantawong et al. (29) created three patches of different composition using an electrospinning method, all of which were made of a combination of silk fibroin (SF) and a synthetic polymer thermoplastic polyurethane (TPU). They implanted each type of patch (*n* = 18) into the abdominal aorta of rats and assessed histopathology at 1, 3, and 6 months after implantation, and concluded that the increase in SF concentration in SF/PU patches had a positive effect on vascular remodeling. Later, Shimada et al. (28) in the team replaced part of the descending aortic wall of the dog with SF/TPU patches for this experiment, and the patches were removed 3 months later for histological examination.

### Surgical Induction of Angiogenesis

Although encouraging results have now been achieved with tissue engineering, the short-term integration of tissue-engineered constructs with the host vasculature remains one of the major obstacles (25). In addition to integrating endothelial cells (49) or angiogenic growth factors (50), surgery-induced angiogenesis appears to be a promising strategy to improve vascularization. In 1980, Erol and Sira (51) demonstrated that neovascularization of the skin through arteriovenous fistula is possible. A vascular bed can be created by use of long inter-positional vein grafts. In 2019, Steiner conducted a similar experiment which resulted that spider silk proteins have good biocompatibility and slow biodegradation, thinner electrospinning fibers showed faster biodegradation and vascularization (25).

## Discussion

### Limitations and Challenges

In the past two decades, through electrospinning technology, cell-loaded nanofiber scaffolds have been widely studied in wound healing (52), drug delivery (53), and cardiac patches (14, 15, 46, 54–56) have shown good performance in preclinical studies of cardiac repair, there are still many problems before clinical implementation (57), such as cardiac patch therapy currently requiring open-heart surgery, which causes anxiety in most patients with myocardial infarction (58), low degree of cell infiltration and cell survival on electrospinning scaffolds (40, 59), insufficient mechanical support performance (60), and biocompatibility that cannot meet clinical needs (61).

The current nanofiber scaffold technology is plagued by some limitations that must be overcome in order to produce highly functional and treatment-related functional engineered cardiac tissues (fECTs), including: (1) Low porosity hinders the deep penetration of seed cells, (2) When cardiomyocytes are cultured on a rigid substrate mimicking a post-infarct fibrotic scar, they lose their synchronized beating, and (3) It is difficult to expand the technology currently used in human applications (62, 63).

In terms of small vessel tissue engineering, Kuang et al. transplanted composite nanofibrous small vessel grafts prepared by a combination of conjugated electrospinning and lyophilization techniques into rabbit carotid arteries (22). Although this team considers matching the degradation rate of vascular stents with the rate of tissue remodeling, it remains a challenge to synchronize the stent degradation rate and new tissue formation rate over a period of time (64, 65).

### Potential Development Direction of Electrospinning

The primary goal of electrospinning technology in cardiovascular tissue regeneration is to prepare good biomimetic scaffolds *in vitro* to regenerate myocardium or vascular tissue and restore their function, and then study their biocompatibility and specific function *in vivo* tests (11, 27).

We may try to do the combination of electrospinning with nanofiber hydrogels (66) to improve their mechanical properties. We can also continue to develop more 3D printed scaffolds as templates to promote cardiomyocyte infiltration (67).

The continuously updated electrospinning technology will certainly promote the development of cardiovascular tissue engineering, such as the melt electrospinning fabricated sinusoidal fibers showing great potential in cardiac tissue regeneration (68, 69).

## Author Contributions

WH wrote the text of this review paper with guidance from RW. NC and MH made valuable suggestions. All authors have reviewed the final version and approved of the content in this manuscript.

## Funding

This work was supported by grants from Military Medicine Youth Special Project of Chinese PLA General Hospital (20QNPY096).

## Conflict of Interest

The authors declare that the research was conducted in the absence of any commercial or financial relationships that could be construed as a potential conflict of interest.

## Publisher's Note

All claims expressed in this article are solely those of the authors and do not necessarily represent those of their affiliated organizations, or those of the publisher, the editors and the reviewers. Any product that may be evaluated in this article, or claim that may be made by its manufacturer, is not guaranteed or endorsed by the publisher.
